# Analysis of the Genetic Diversity and Genetic Structure of Jiangshan Black Pigs Using Single Nucleotide Polymorphism (SNP) Chips

**DOI:** 10.3390/ani14182660

**Published:** 2024-09-12

**Authors:** Mingfei Zhu, Litong Wang, Zhibo Ma, Yangcang Mao, Guoshui Wang, Rong Zheng, Bo Zuo, Yizhen Wang

**Affiliations:** 1 Key Laboratory of Molecular Animal Nutrition, Ministry of Education, College of Animal Sciences, Zhejiang University, Hangzhou 310058, China; wh60606060@163.com; 2 Zhejiang Tianpeng Group Co., Ltd., Jiangshan 324100, China; tp8880999@163.com (L.W.); jsxmj999@126.com (Y.M.); wgs67072323@163.com (G.W.); 3 Key Laboratory of Agriculture Animal Genetics, Breeding and Reproduction of the Ministry of Education, Huazhong Agricultural University, Wuhan 430072, China; mzb0000109@163.com (Z.M.); zhengrong@mail.hzau.edu.cn (R.Z.)

**Keywords:** genetic diversity, genetic structure, inbreeding coefficient, Jiangshan black pig, SNP chips

## Abstract

**Simple Summary:**

This study investigated the genetic attributes of the Jiangshan black pig conservation population, with a specific focus on genetic diversity, family structure, and levels of inbreeding. The results provide significant insights for the management and conservation of a pig population with limited numbers. The study proposes the use of genomic data by breeders to improve breeding practices, especially by taking into account familial relationships. Suggestions include maintaining an equitable distribution of male and female pigs within each family and choosing individuals with a kinship coefficient lower than 0.1 for breeding purposes.

**Abstract:**

The Jiangshan black pig is an indigenous pig breed in China, renowned for its superior meat quality and adaptability to roughage. However, the population of Jiangshan black pigs has dwindled due to the introduction of cosmopolitan pig breeds and the outbreak of African swine fever, putting them at risk of extinction. To offer insights into the conservation and breeding of Jiangshan black pigs, this study involved 118 Jiangshan black pigs as the research subjects and employed the Zhongxin-1 Porcine Breeding Array PLUS chip to detect whole-genome SNPs. Additionally, various software tools were utilized to accurately analyze the genetic diversity, phylogenetic relationship, inbreeding coefficient, and pedigree structure of the Jiangshan black pig conservation population. The findings indicated that the effective population size (Ne) of the Jiangshan black pig was 4.9, with an average inbreeding coefficient of 0.253 within the population. A genetic relationship analysis identified that the 16 male pigs were clustered into four families, and the 100 female pigs were also assigned to these familial groups. Furthermore, two female pigs were classified as “other” due to their distant genetic relatedness to all the males. These results contribute to a better understanding of the current status of the conservation of the Jiangshan black pig population and offer a theoretical foundation for the development of conservation strategies.

## 1. Introduction

The Jiangshan black pig is a notable local pig breed in the Zhejiang Province in China, primarily found in and around Jiangshan City. This breed is known for its ability to thrive on coarse feed and high meat quality. The high quality of the meat is characterized by a small muscle fiber diameter of 36.75 µm, a high muscle fiber density of 512.86 fibers/mm^2^, a moderate intramuscular fat content of 4.30%, and a significant proportion of unsaturated fatty acids, particularly polyunsaturated fatty acids, which account for 14.08% in castrated boars and 12.92% in sows. These exceptional attributes contribute to the palatability of pork, which remains flavorful even when prepared by boiling it in water without the addition of salt [1]. The breeding history of Jiangshan black pigs can be traced back over 1600 years, as evidenced by cultural artifacts, including pottery pig pens from the Eastern Jin Dynasty. [2]. In 1974, the East China Pig Breeding Cooperation Group designated the Jiangshan black pig, along with the Guangfeng black pig and Yushan black pig, as the Yujiang pigs. The information was incorporated into the Chinese Pig Breeds Chronicles in 1986.. The population of Jiangshan black pigs peaked in the early 1980s. In 1980, there were 16,877 pigs, including 199 breeding pigs. However, after the mid-1980s, China began importing pig breeds like the Yorkshire, Landrace, and Duroc, which are known for their rapid growth and efficient feed-to-meat conversion rates [3]. This influx of new breeds led to a decline in the Jiangshan black pig population, and this downward trend continued throughout the 1990s [2]. In addition, the outbreak of African swine fever in recent years has further worsened the situation of the Jiangshan black pigs. According to a survey, there were 200 Jiangshan black pigs in 2018, 100 in 2019, and 60 in 2020 [4]. However, since 2021, the population has increased to 580 due to the implementation of protective measures. The conservation population of Jiangshan black pigs was sourced from local farmers, but due to unclear pedigree information, challenges have arisen in understanding the genetic structure and diversity of the population. This has posed obstacles to breeding, development, and utilization efforts. To address these issues, this study employs chip analysis to conduct a comprehensive genetic investigation aimed at elucidating the genetic characteristics of the Jiangshan black pig conservation population.

Chip analysis is a contemporary molecular genetic technique that employs gene chip technology to identify and compare genetic markers across various DNA loci. This method can unveil the genetic structure and diversity of species and populations [5,6]. By utilizing this approach, researchers can elucidate the genetic relationships among individuals, assess inbreeding levels, and evaluate the genetic diversity of the Jiangshan black pig conservation population. The outcomes of this analysis not only address the existing gaps in population genetics knowledge related to the Jiangshan black pig but also hold significant implications for devising appropriate conservation strategies, enhancing breeding practices, and optimizing population management and utilization.

## 2. Materials and Methods

### 2.1. Animals

In accordance with the principle that the tested individuals should represent all lineages of the population, a total of 118 Jiangshan black pigs were selected for blood sampling at the Jiangshan Black Pig Breeding Farm of the Zhejiang Tianpeng Group, including 16 boars and 102 sows. The blood samples obtained were placed in anticoagulant tubes and stored in a low-temperature refrigerator.

### 2.2. DNA Extraction and Quality Control

A DNA sample was isolated utilizing the Cwbio CW2531Q Blood DNA Extraction Kit [7], and the optical density (OD) values of the genomic DNA were assessed at 260 nm and 280 nm wavelengths using the UV spectrophotometer NanoDrop 2000 (Thermo Scientific, Waltham, MA USA). The 260/280 ratio needed to fall within the range of 1.7 to 2.1, with an individual sample concentration exceeding 50 ng/μL.

### 2.3. Chip Detection

After the test, the DNA sample underwent testing using the Zhongxin-1 Porcine Breeding Array PLUS chip (Beijing Compass Agricultural Technology Co., Ltd., Beijing, China) for SNP genotyping. Subsequently, the genotype data were subjected to quality control using Plink (V1.90) software [8]. Following quality control procedures (i. retaining the SNPs located on autosomes; ii. removing the SNPs with a minimum allele frequency (MAF) below 0.01; iii. removing the SNPs with a call rate less than 0.90; and iv. removing the SNPs with a Hardy–Weinberg equilibrium *p*-value less than 1 × 10^−6^), only sites with optimal typing quality were preserved for further analysis [9].

### 2.4. Genetic Diversity Analysis

The analysis of genetic diversity using PLINK software (v1.90) involves evaluating multiple parameters, including Ne, the polymorphic marker ratio (P_N_), the expected heterozygosity (H_E_), the observed heterozygosity (H_O_), the polymorphic information content (PIC), the number of effective alleles (Ae) and MAF.

Ne refers to the ideal population content that has the same gene frequency variance or inbreeding coefficient increment (heterozygosity decay rate) as the actual population [10]. It is usually estimated based on the level of linkage disequilibrium (LD) in the population [11]. The formula utilized for its calculation is as follows: Ne = (1/4c) × (1/r^2^ − 1) [12,13,14], where c denotes the molar distance between SNP sites in millimeters, and r^2^ signifies the degree of linkage between SNP sites.

P_N_ refers to the proportion of polymorphic loci in the target population to the total number of loci. Initially, the MAF for each locus is computed using the Plink software [8], followed by the calculation of P_N_ using a custom R script. P_N_ is determined using the formula P_N_ = M/N, where M denotes the count of polymorphic loci and N represents the total number of loci.

H_E_ is the ratio of heterozygosity at any site across all individuals, and H_O_ is the ratio of individuals where a site is heterozygous compared to all individuals [10]. Discrepancies between H_E_ and H_O_ can suggest different genetic influences within a population. If H_E_ is less than H_O_, the population may be influenced by migration or gene flow; while if H_E_ is more than H_O_, selection or inbreeding may have occurred in the population [10]. The calculation of H_E_ and H_O_ was performed using PLINK software version 1.90.

Ae refers to the number of alleles required at a locus in an ideal population (where all alleles have equal frequencies) to produce homozygosity similar to that in the actual population. It is equal to the reciprocal of the homozygosity of the actual population.

MAF is the minimum allele frequency, which usually refers to the frequency of uncommon alleles in a given population, such as TT, TC, and CC genotypes. If the frequency of C in the population is 0.36 and T is 0.64, then allele C is the minimum allele, and the MAF is 0.36.

### 2.5. Genetic Relationships and Population Structure Analysis

We utilized the Plink software version 1.90 to calculate the genetic distance of identity by state (IBS) among individuals. We employed the G matrix software version 2 to construct a population G matrix and generate a heatmap to illustrate the genetic relatedness among pigs [15]. Using the Neighbor Joining (NJ) method in Mega X for population clustering analysis, we analyzed the population structure of Jiangshan black pigs [16]. Additionally, we quantified the distribution of male and female pigs across various familial groups.

### 2.6. Inbreeding Coefficient Analysis

We utilized Plink version 1.90 to calculate the length of the runs of homozygosity (ROH) on the genome of each sample [8]. Subsequently, the ratio of the cumulative length of the ROH segments to the total length of the autosomal genome (approximately 2,449,672.641 kb in pig) was assessed to derive the inbreeding coefficient associated with ROH [17]. The mean inbreeding coefficient for the population was computed by aggregating the individual inbreeding coefficient values and dividing the sum by the total number of individuals in the sample set.

## 3. Results

### 3.1. Genotype Quality Control

**Figure 1:**
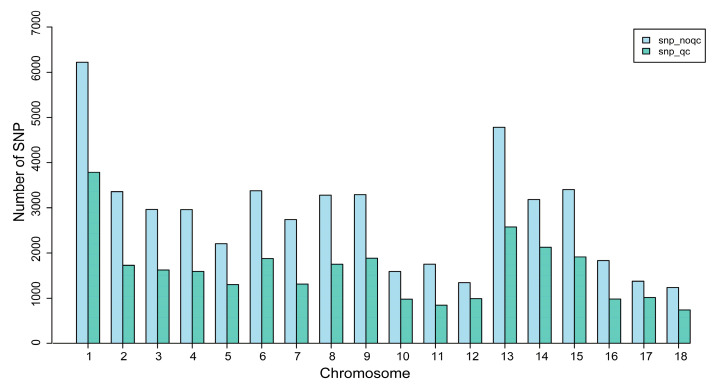
Distribution of SNPs on each chromosome before and after quality control. The abscissa represents the chromosome number, and the ordinate represents the number of SNPs.

Following the extraction and qualification of genomic DNA from Jiangshan black pigs, genotyping of the samples was conducted using the “Zhongxin-1” gene chip. The quality of the genotype data was managed using the Plink (v1.90) software. Only loci meeting the standard genotyping quality were retained for subsequent analysis. The outcomes indicated that 29,033 SNPs passed the quality control (Table 1). The chromosome 1 had the highest number of SNPs with 3786, while chromosome 18 had the lowest with 743 (Figure 1). This suggests that there is likely significant genetic diversity present in the genome of Jiangshan black pigs, particularly characterized by a high density of SNPs on chromosome 1. This finding offers valuable insights for further investigations into the genetic traits of Jiangshan black pigs, as well as their potential applications in breeding and conservation efforts.

### 3.2. Genetic Diversity

**Figure 2:**
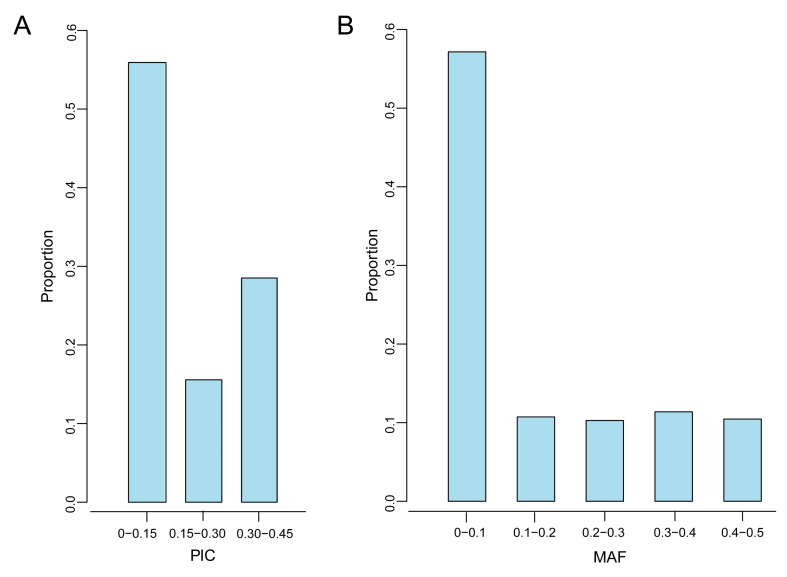
(**A**) Distribution of PIC. The abscissa represents the PIC interval value, and the ordinate represents the SNP proportion. (**B**) Distribution of MAF. The abscissa represents the MAF interval, and the ordinate represents the SNP proportion.

The genetic diversity of the Jiangshan black pig conservation population was analyzed using Plink (v1.90) software (Table 2). The Ne was determined to be 4.9. The P_N_ at the SNP loci was found to be 0.507, which suggested a relatively rich genetic diversity within the Jiangshan black pig population. The H_O_ (0.345) slightly exceeded the H_E_ (0.315), which indicated differentiation within the conservation population. The PIC of SNPs was 0.147, with 16,913 SNPs identified as moderately polymorphic (PIC > 0.25). Specific distribution ranges can be observed in Figure 2A. The MAF ranged from 0 to 0.5, with an average of 0.137. The largest proportion fell within the range of 0–0.1, accounting for 57.16%, while the remaining frequencies were evenly distributed (Figure 2B). The findings suggest that the protected population of Jiangshan black pigs exhibits significant genetic diversity. Despite the small effective population size, it offers a robust genetic foundation for future breeding and conservation efforts. This discovery serves as a valuable reference for comprehending the genetic characteristics of this population and its potential role in preserving biodiversity.

### 3.3. Analysis of Kinship

**Figure 3:**
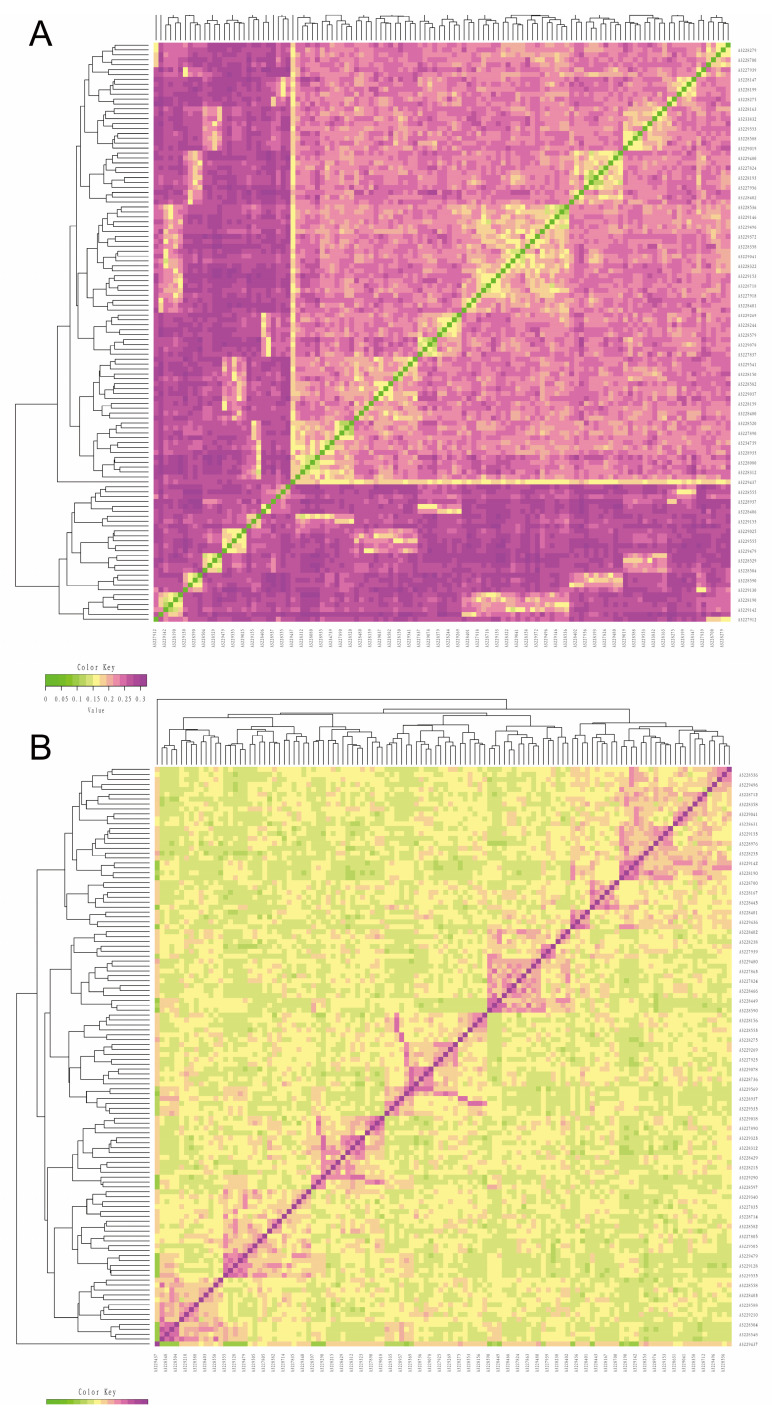
(**A**) Visualization results of the genetic relationship analysis based on the IBS distance matrix. The figure illustrates the genetic distance between two individuals, with color proximity indicating a closer genetic relationship. The individual ID is represented by the vertical and horizontal coordinates. (**B**) Visualization results of the genetic relationship analysis based on the G matrix. The figure illustrates the coefficient of the genetic relationship between two individuals, where the color proximity indicates the closeness of the genetic relationship. The abscissa and ordinate axes represent the individual IDs.

We utilized Plink software version 1.90 to compute the IBS genetic distance between individuals for the analysis of the genetic relatedness within the Jiangshan black pig population (Figure 3A). The IBS genetic distance distribution range within the Jiangshan black pig population spans from 0.1089 to 0.3201, with an average IBS genetic distance of 0.2418. The majority of individuals exhibit relatively long IBS genetic distances, indicating a moderate level of genetic relationship (depicted in purple); however, some individuals display close IBS genetic distances (depicted in yellow), suggesting a high risk of inbreeding within this subgroup. Subsequently, the genetic relatedness of the Jiangshan black pig conservation population was further confirmed through the utilization of the genomic relationship G matrix established by the SNP loci (Figure 3B). The genetic relationship findings derived from the G matrix align with those obtained from the IBS genetic distance matrix, suggesting that certain individuals within the Jiangshan black pig conservation population exhibit a notable genetic affinity (depicted in the purple section). The results underscore the genetic complexity present within the protected population of Jiangshan black pigs and indicate the potential risks associated with inbreeding. These findings hold significant reference value for the formulation of genetic management and conservation strategies, which may enhance efforts to preserve the genetic diversity of the population.

### 3.4. Inbreeding Coefficient

**Figure 4:**
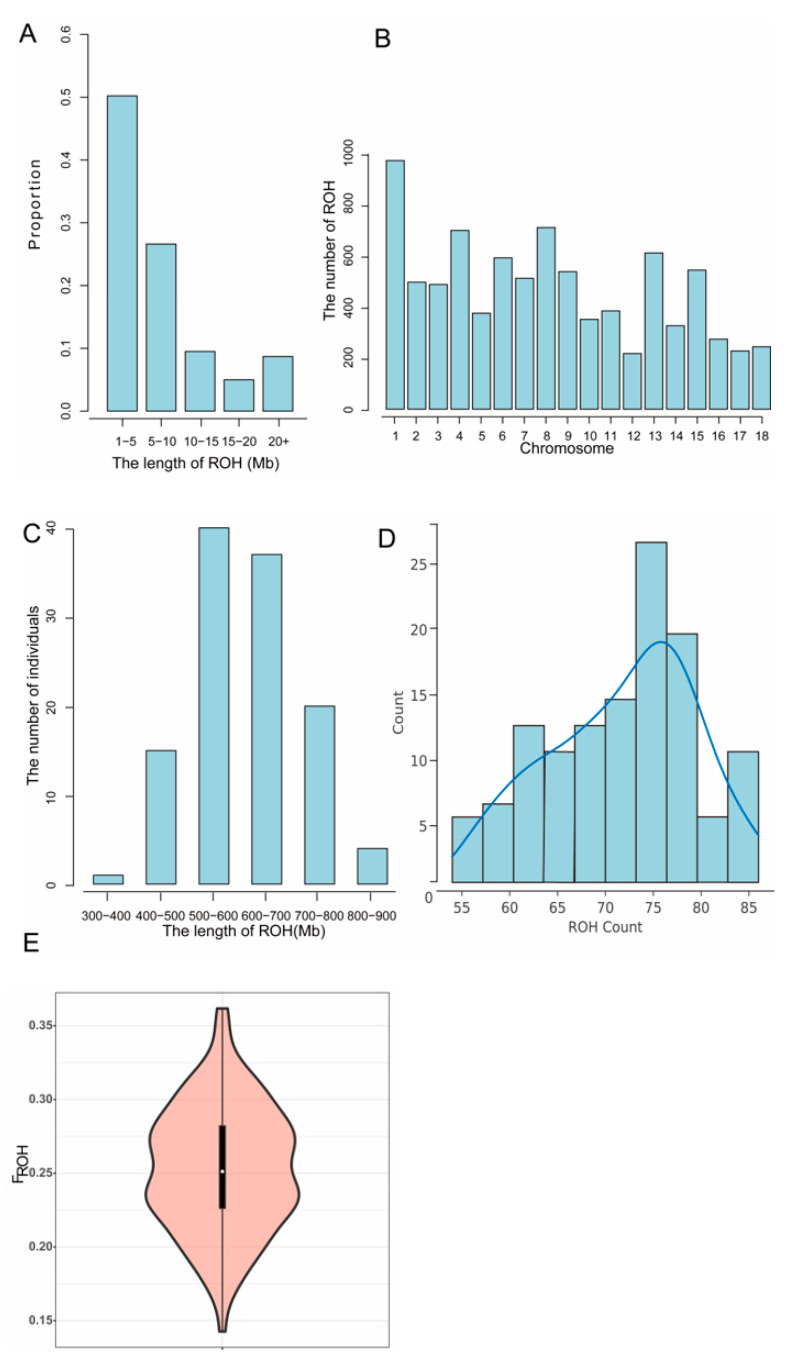
(**A**) Distribution of ROH length in Jiangshan black pig population. (**B**) Distribution of ROH quantity on each chromosome in Jiangshan black pig population. (**C**) Distribution of ROH sample numbers in Jiangshan black pigs. The abscissa represents the length interval of ROH, and the ordinate represents the number of individuals. (**D**) Distribution of ROH quantities in the Jiangshan Black Pig Population. (**E**) Distribution ratio of inbreeding coefficient (F_ROH_) based on ROH in Jiangshan black pigs. The violin plot is primarily utilized to illustrate the distribution of data. The central white dot represents the median of the group, and the upper and lower edges of the black box in the middle correspond to the upper and lower quartiles of the group, respectively. The width of the violin plot indicates the probability density distribution of the group, a wider violin plot suggests a higher number of samples at that level, and conversely, a narrower plot indicates fewer samples.

Utilizing Plink software version 1.90 for the detection of the number and length of ROH in the Jiangshan black pig population, a total of 8534 ROH fragments were identified. The findings revealed that the least number of ROH fragments, constituting 4.99%, were observed in the 15–20 Mb range, while the highest number, accounting for 50.21%, were found in the 1–5 Mb range (Figure 4A). The length distribution of these ROH fragments indicates that the population may have undergone a greater degree of inbreeding in the past, leading to a higher prevalence of the shorter homologous segments. Chromosome 1 exhibited the highest number of ROH fragments, with 969, whereas chromosome 12 displayed the lowest count at 217. The distribution of ROH fragments across other chromosomes was relatively consistent (Figure 4B). This distribution may be associated with a higher prevalence of the genetic variations on chromosome 1, indicating the potential presence of significant genetic characteristics within these regions. The total length of ROH in each Jiangshan black pig ranged from 349.92 to 886.13 Mb, with an average total length of 619.84 Mb. Notably, the majority of individuals had ROH lengths between 500 and 600 Mb, representing 33.61% of the population (Figure 4C). In addition, the population distribution of ROHs was relatively skewed, with a greater number of individuals exhibiting higher ROH counts (Figure 4D). This pattern suggests that the population has experienced inbreeding. The range of inbreeding coefficients, which spans from 0.1428 to 0.3617, with a mean value of 0.253, further substantiates this perspective (Figure 4E). In conclusion, the analysis of ROH and the assessment of inbreeding coefficients have highlighted the potential genetic risks present within the Jiangshan black pig population. The accumulation of inbreeding may lead to genetic defects and reduced adaptability. Consequently, it is imperative to monitor inbreeding levels and implement appropriate management strategies, such as the introduction of external genetic material and the optimization of breeding practices, to preserve the genetic diversity of this population.

### 3.5. Cluster Analysis and Family Construction

**Figure 5:**
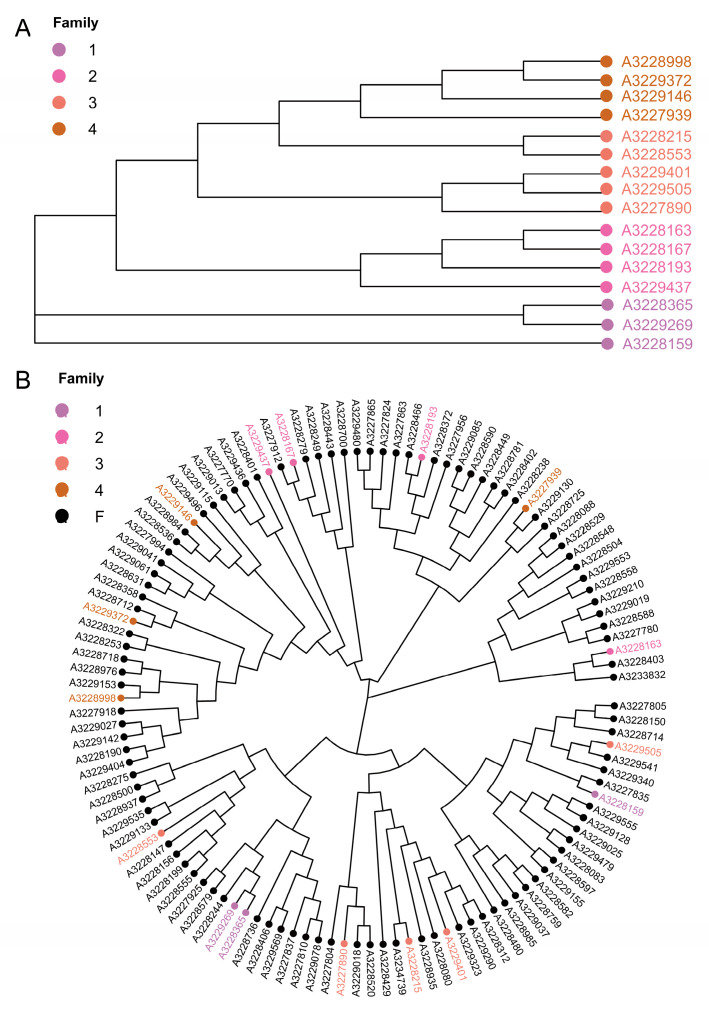
(**A**) Cluster analysis results for boars in Jiangshan black pig population. (**B**) Cluster analysis results for all Jiangshan black pigs. The color in the evolutionary tree is the boar sample, and one color represents a family.

In consideration of the significance of boars within the conservation population, this study initially employed a genetic relationship coefficient of ≥0.1 among boars as the criterion. The NJ method with Mega X was utilized to cluster the boar population to assess their genetic relationships. The outcomes found that sixteen boars were categorized into four families (Figure 5A). Subsequently, based on the kinship level between each sow and boars from different families, the sows were allocated into four boar families (Figure 5B). The findings revealed that, apart from the four boar-containing families, the Jiangshan black pig conservation population also included two sows that were genetically distant from all the boars, thus being classified under the “other” category. The quantity of boars and sows in each family is detailed in Table 3. Sows in certain families were interbred and distributed across several distinct families. For instance, sow a3227925 was simultaneously assigned to families 1, 2, 3, and 4. These findings underscore the complex nature of the family structure within the Jiangshan black pig conservation population, as well as the mating patterns of sows across various boar families. This observation serves as a critical foundation for developing more effective genetic management and breeding strategies aimed at preserving the genetic diversity and overall health of the population.

## 4. Discussion

The Jiangshan black pig conservation population is characterized by a small size, and historical pedigree records are incomplete. The lack of knowledge regarding genetic relationships may lead to inadvertent inbreeding, consequently diminishing the genetic diversity within the conservation population. Therefore, the application of modern gene analysis technology is imperative for the systematic evaluation of the Jiangshan black pig breed to facilitate informed conservation efforts. SNP chip technology enables the simultaneous detection and analysis of thousands to millions of SNP loci, unveiling genotype variations among distinct individuals. This technology is extensively employed in genetics, population genetics, genetic relationship analysis, gene association studies, and genetic enhancement strategies, offering an efficient, precise, and high-throughput approach to comprehensively understanding the genetic attributes and evolutionary history of organisms [18,19]. The “ Zhongxin-1” chip has over 50,000 SNP loci. It is not only suitable for international pig breeds, but also for the genome analysis of Chinese local pigs, with high-efficiency and accuracy advantages [18,20,21,22]. Therefore, in this investigation, the genetic diversity, genetic relationships, inbreeding coefficient, and family structure of the Jiangshan black pig population were assessed using the “Zhongxin-1” chip. This analysis provided essential data to support the protection, advancement, and sustainable utilization of the Jiangshan black pig population.

The analysis revealed that the Ne of the Jiangshan black pig was 4.9, which was lower when compared with other local pig conservation populations in China, such as the Licha black Pig (8.7) [23], Pudong white pig (45) [24], Tunchang pig (73) [25], Liangshan pig (15) [19], and Tongcheng pig (14) [5], but slightly higher than the Rongchang pig (3.2) [22] and Hechuan black pig (4.2) [26]. A lower Ne suggests reduced genetic diversity, which could potentially lead to the accumulation of genetic defects, an increased risk of extinction, and compromised genetic health [27]. Heterozygosity is categorized into H_O_ and H_E_. By comparing the differences between H_O_ and H_E_, the genetic structure and evolutionary trend within the population can be elucidated. Consistent with findings from various local pig breeds in China, including the Qingni Black Pig [28] and Hechuan black pig [26], this study observed that H_O_ was slightly higher than H_E_, indicating some degree of outbreeding within the population. Additionally, parameters such as P_N_, PIC, Ae, and MAF were examined to assess gene polymorphism from various perspectives. When compared to other local pig breeds in China, such as the Tongcheng pig [5], Liangshan pig [19], Rongchang pig [22], Hechuan black pig [26], and Taihu pig [29], the indicators for the Jiangshan black pig were found to be at moderate to low levels, suggesting relatively low genetic diversity and limited polymorphism information. To preserve the genetic health and diversity of the population and ensure effective conservation efforts, it is recommended that focus be put on enlarging the conservation population, enhancing gene flow to boost Ne, promoting inter-population communication, introducing new bloodline genes sensibly to increase genetic diversity, implementing appropriate reproductive management to mitigate inbreeding effects, and reinforcing genetic monitoring and management practices to uphold the genetic well-being of the population [29].

ROH represents extended homozygous segments within the genome. The varying lengths of ROH are linked to the genetic backgrounds of individuals and shared ancestors. As genetic relatedness decreases, the length of ROH segments also decreases. ROH contains a wealth of genetic information that can be utilized to assess inbreeding levels and genetic diversity, infer population structure and history, as well as identify and screen the functional genes associated with economically important traits [30,31,32]. This study explores the inbreeding levels and family structures within the Jiangshan black pig conservation population through an analysis of inbreeding coefficients and a cluster analysis based on ROH. The results indicate that the inbreeding coefficient within the Jiangshan black pig population ranged from 0.1428 to 0.3617, with an average inbreeding coefficient of 0.253. Which is higher than that of the Tongcheng pig [5], Hechuan black pig [26], and Taihu pig [29]. The elevated inbreeding coefficient suggests the presence of some degree of inbreeding among individuals in the population [33]. Through cluster analysis and family construction, 16 boars were categorized into four families. Furthermore, based on the genetic relationships between the sows and boars from the different families, two sows were identified within the Jiangshan black pig conservation population with distant blood relationships from these known families. These results underscore the importance of closely monitoring the level of inbreeding and the family structure within the Jiangshan black pig population. A higher level of inbreeding could potentially lead to a decrease in genetic diversity and the accumulation of genetic defects. To safeguard the genetic health and diversity of the Jiangshan black pig, it is essential to maintain a balanced population comprising individuals from various lineages [34]. The subsequent breeding and selection of breeding pigs should adhere to the following guidelines: Firstly, individuals with distant kinship relationships should be selected for mating based on the coefficient of kinship, thereby minimizing the breeding of boars and sows within the same familial lineage. Additionally, given the relatively high overall level of inbreeding within the population, it is advisable to expand the search area to identify new individuals of Jiangshan black pig or similar breeds. This approach aims to enhance genetic diversity within the population and mitigate the escalation of inbreeding. Furthermore, conducting annual monitoring of the population’s genetic diversity and inbreeding status to clarify conservation efforts and further reduce inbreeding rates is recommended. It is particularly important to protect and manage two sows that possess significant genetic contributions due to their distant blood relationships with the established families; these sows should be allowed to mate with either boar to preserve their valuable genetic diversity. Lastly, the establishment of a frozen sperm bank for breeding boars should be prioritized to prevent the loss of genetic resources in the event of mortality due to diseases or other unforeseen circumstances.

## 5. Conclusions

This study employed chip detection technology to assess the genetic diversity, genetic relationships, inbreeding coefficient, and family structure within the protected population of Jiangshan black pigs. The findings indicate a significant level of genetic diversity within this population. However, the effective population size is constrained, characterized by a limited number of families and substantial disparities in the number of individuals per family, which contributes to an increase in inbreeding. Consequently, it is imperative to implement measures to mitigate inbreeding levels. Such measures may include the utilization of boars and sows from distinct familial lineages for reproduction, the introduction of new family genes, and the judicious management of individual numbers within each family to ensure a balanced family structure. These results offer a theoretical foundation for the formulation of conservation strategies for Jiangshan black pigs and serve as a reference for the protection of species with small populations.

## Figures and Tables

**Table 1:** SNP quality control statistics.

Quality Control Standards	Number of SNPs Tags
Total number of SNPs	57,466
SNP with MAF < 0.01	20,888
SNP noting Hardy–Weinberg equilibrium *p* < 10^−6^	147
SNP with call rate < 0.90	830
SNPs on chromosome X	4252
SNPs on chromosome 0	2310
Insertion/deletion	6
SNPs used after quality control	29,033

**Table 2:** Genetic diversity parameters of the Jiangshan black pig conservation population.

Genetic Diversity Parameters	Parameter
Ne	4.9
P_N_	0.507
H_E_	0.315
H_O_	0.345
PIC	0.147
Ae	1.528
MAF	0.137

**Table 3:** Family Construction statistical Results of Jiangshan black pigs.

Family	Sex	Amount
1	boar	5
	sow	64
2	boar	13
	sow	99
3	boar	9
	sow	84
4	boar	7
	sow	78
other	sow	2

## Data Availability

The data analyzed during the current study are available from the corresponding author upon reasonable request.
